# Machine learning for predicting pathological complete response in patients with locally advanced rectal cancer after neoadjuvant chemoradiotherapy

**DOI:** 10.1038/s41598-020-69345-9

**Published:** 2020-07-28

**Authors:** Chun-Ming Huang, Ming-Yii Huang, Ching-Wen Huang, Hsiang-Lin Tsai, Wei-Chih Su, Wei-Chiao Chang, Jaw-Yuan Wang, Hon-Yi Shi

**Affiliations:** 1 Department of Radiation Oncology, Kaohsiung Medical University Hospital, Kaohsiung, Taiwan; 2 Department of Radiation Oncology, Faculty of Medicine, College of Medicine, Kaohsiung Medical University, Kaohsiung, Taiwan; 3 Department of Radiation Oncology, Kaohsiung Municipal Ta-Tung Hospital, Kaohsiung Medical University, Kaohsiung, Taiwan; 4 Graduate Institute of Medicine, College of Medicine, Kaohsiung Medical University, Kaohsiung, Taiwan; 5 Center for Cancer Research, Kaohsiung Medical University, Kaohsiung, Taiwan; 6 Division of Colorectal Surgery, Department of Surgery, Kaohsiung Medical University Hospital, Kaohsiung Medical University, No. 100, Tzyou 1st Road, Kaohsiung, 807 Taiwan; 7 Department of Surgery, Faculty of Medicine, College of Medicine, Kaohsiung Medical University, Kaohsiung, Taiwan; 8 Graduate Institute of Clinical Medicine, College of Medicine, Kaohsiung Medical University, Kaohsiung, Taiwan; 9 School of Pharmacy, Taipei Medical University, Taipei, Taiwan; 10 Department of Healthcare Administration and Medical Informatics, Kaohsiung Medical University, 100, Shih-Chuan 1st Road, 80708 Kaohsiung, Taiwan; 11 Department of Business Management, National Sun Yat-Sen University, Kaohsiung, Taiwan; 12 Deoartment of Medical Research, Kaohsiung Medical University Hospital, Kaohsiung, Taiwan; 13 Department of Medical Research, China Medical University Hospital, China Medical University, Taichung, Taiwan

**Keywords:** Cancer, Computational biology and bioinformatics, Gastroenterology, Oncology

## Abstract

For patients with locally advanced rectal cancer (LARC), achieving a pathological complete response (pCR) after neoadjuvant chemoradiotherapy (CRT) provides them with the optimal prognosis. However, no reliable prediction model is presently available. We evaluated the performance of an artificial neural network (ANN) model in pCR prediction in patients with LARC. Predictive accuracy was compared between the ANN, *k*-nearest neighbor (KNN), support vector machine (SVM), naïve Bayes classifier (NBC), and multiple logistic regression (MLR) models. Data from two hundred seventy patients with LARC were used to compare the efficacy of the forecasting models. We trained the model with an estimation data set and evaluated model performance with a validation data set. The ANN model significantly outperformed the KNN, SVM, NBC, and MLR models in pCR prediction. Our results revealed that the post-CRT carcinoembryonic antigen is the most influential pCR predictor, followed by intervals between CRT and surgery, chemotherapy regimens, clinical nodal stage, and clinical tumor stage. The ANN model was a more accurate pCR predictor than other conventional prediction models. The predictors of pCR can be used to identify which patients with LARC can benefit from watch-and-wait approaches.

## Introduction

Neoadjuvant chemoradiotherapy (CRT) has benefited patients with locally advanced rectal cancer (LARC) with specific respect to improvements in local control, disease-free survival, and sphincter preservation rates^1–3^. However, the patterns of tumor regression after neoadjuvant CRT vary widely, ranging from a pathological complete response (pCR) to disease progression. Patients with a pCR have the most favorable survival and tumor control, but only 10–30% of patients with LARC achieve a pCR to neoadjuvant CRT^4–7^. Furthermore, mounting evidence has demonstrated that in patients who achieve a pCR, radical surgery can be omitted without compromising tumor control^8,9^. Therefore, the identification of useful predictors of a pCR in patients with LARC after neoadjuvant CRT is vital.

Few studies have compared the artificial neural network (ANN), *k*-nearest neighbor (KNN), support vector machine (SVM), naïve Bayes Classifier (NBC), and multiple logistic regression (MLR) models with respect to internal validity (reproducibility). Validity is a crucial performance metric^10,11^. However, numerous predictive models yield insufficiently reliable predictions of pCR occurrence in patients with LARC after neoadjuvant CRT.

One of the most frequently applied methods for multivariate analysis is regression analysis; in this type of analysis, linear correlations between dependent and independent variables are assumed. Studies have demonstrated that biomedical variables usually vary nonlinearly^12–16^. The KNN model is a simple classification algorithm with straightforward implementation^14^. The KNN model predicts new samples by using training samples; the process entails a majority vote on the outcome of points that are k-nearest to the new sample. The SVM model is a supervised learning model associated with learning algorithms that analyse information used for regression analysis and classification^13^. An SVM model constructs multidimensional hyperplanes that separate the 2 classes while maximizing the margin between the 2 classes; it uses kernel functions and can discriminate between nonlinearly separable classes. An NBC model can be used to efficiently develop classification tools for various health domains and transform complex clinical problems into clear, precise, and predictive models^16^. An ANN model has three layers: input, hidden, and output layers. Every layer has nodes connected by links from one layer to the next^12,15^. Nodes in the input layer represent predictors, whereas those in the output layer are considered outcome variables. A common application of neural networks is the multilayer backpropagation learning algorithm, which models nonlinear systems. Although the interpretation of neural networks is more complicated than that of other statistical models, the ANN model has been used in various medical fields.

Although considerable improvements in outcome prediction models have been achieved, pCR prediction models continue to have major limitations^17,18^. For example, many studies have identified effective pCR predictors, but most related variables have exhibited insufficient sensitivity and specificity^19–21^. Therefore, in our study, ANN, KNN, SVM, NBC, and MLR models were used to identify the most powerful predictors of pCR in patients with LARC after neoadjuvant CRT. Thus, the primary purpose of this study was to validate the accuracy of the ANN model for pCR prediction in patients with LARC following neoadjuvant CRT. The secondary purpose was to investigate the predictive performance of various forecasting models.

## Methods

### Patients

**Figure 1:**
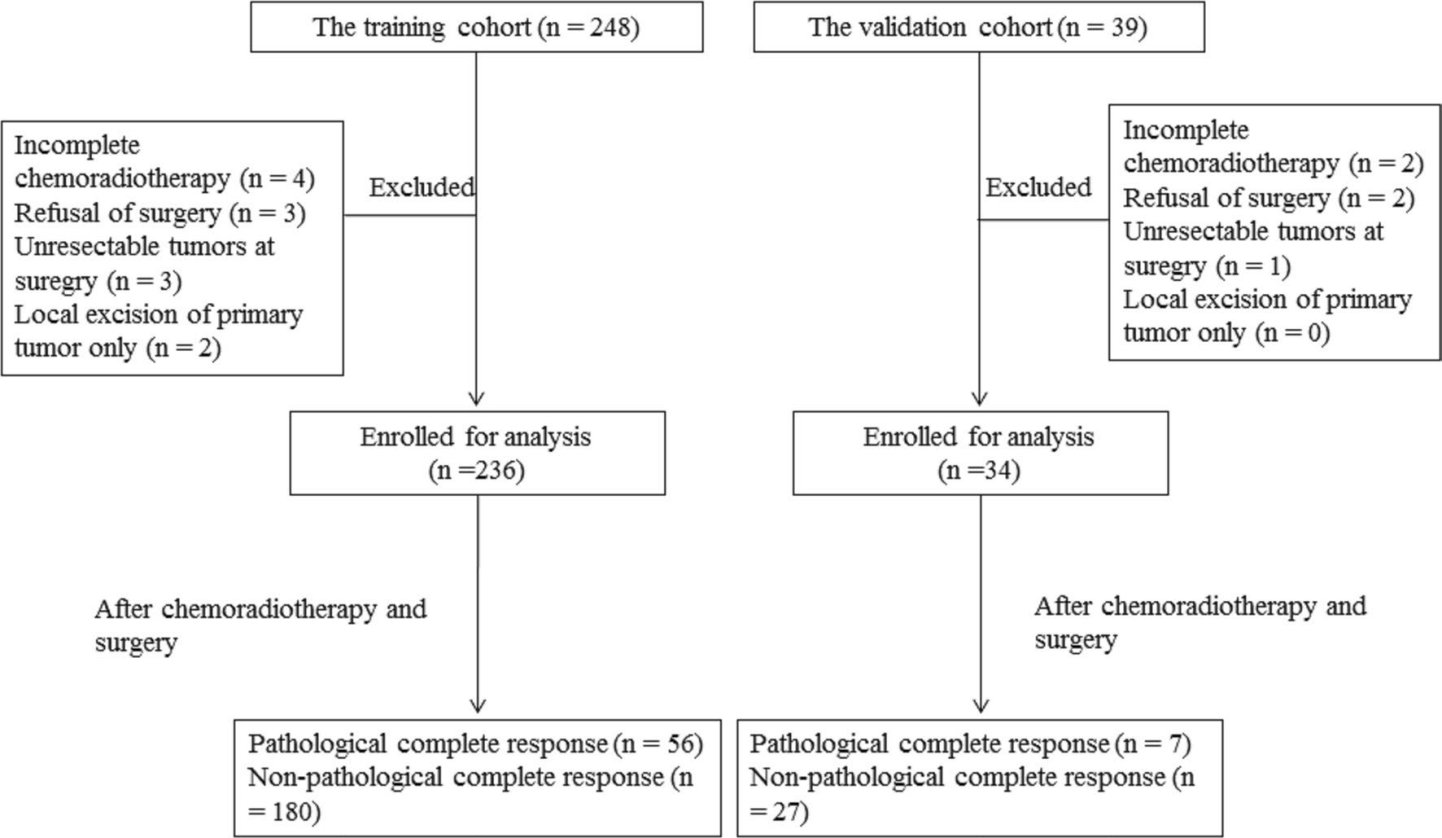
Flow chart of patient selection for the training and validation cohorts.

This study identified patients with a LARC diagnosis who were undergoing neoadjuvant CRT at any period between January 2011 and December 2017 at Kaohsiung Medical University Hospital. In total, 248 consecutive patients satisfied the inclusion criteria, which were pathologically proven adenocarcinomas, tumors located within 12 cm of the anal verge, clinical stage II and III rectal tumors (T3 to 4 or N +), and the delivery of neoadjuvant CRT. We excluded twelve patients because they had incomplete neoadjuvant CRT (n = 4), rejection of resection (n = 3), unresectable tumors after CRT (n = 3), or only primary tumor excision (n = 2). The remaining 236 patients were enrolled for analysis as the training cohort. For the validation cohort, 34 patients with LARC were recruited at Kaohsiung Medical University Hospital between January 2018 and September 2018. The same inclusion and exclusion criteria were used for the training and validation cohorts (Fig. 1). Pretreatment clinical staging was determined through computed tomography (CT) of the abdomen and chest, pelvic magnetic resonance imaging (MRI), and a physical examination. Participants’ serum carcinoembryonic antigen (CEA) levels and routine laboratory test results were analyzed.

### Treatment

All participants underwent neoadjuvant CRT. Radiotherapy was delivered from 45 to 50.4 Gy, 1.8 to 2.0 Gy per fraction. Three-dimensional conformal radiotherapy was administered to 45 patients, and intensity-modulated radiotherapy was administered to 191 patients. Chemotherapy was administered concurrently with radiotherapy. Participants underwent 1 of the following 2 chemotherapeutic regimens. The first was the fluoropyrimidine-based regimen (n = 95), which consisted of six courses of capecitabine (850 mg/m^2^ twice daily for 14 days) followed by 7 days of rest after each course. The second was a biweekly schedule of FOLFOX, which included oxaliplatin (85 mg/m^2^) on day 1, in addition to folinic acid (400 mg/m^2^) and a 46-h infusion of 5-fluorouracil (2,800 mg/m^2^) repeated every 2 weeks during radiotherapy; patients continued to receive three to four cycles of consolidation chemotherapy with biweekly FOLFOX after completion of radiotherapy (n = 141)^7^.

All patients in the current study underwent total mesorectal excision after completing neoadjuvant CRT. The surgical procedures included low anterior resection with colorectal or coloanal anastomosis (n = 207) and abdominoperineal resection (n = 29).

### Evaluation and follow-up

Two experienced pathologists evaluated tumor responses to neoadjuvant CRT. A pCR was defined as the absence of malignant cells in primary tumors and nodes (ypT0N0) in the resected specimen following neoadjuvant CRT.

Acute side effects were assessed at each visit during neoadjuvant CRT according to the Common Terminology Criteria for Adverse Events, version 4.03. We defined anemia as a hemoglobin level of < 10 g/dL. Approximately 6–10 weeks after completing CRT, measurements were conducted before surgery, specifically through pelvic MRI, abdominal and chest CT, a CEA test, and colonoscopy. After treatment completion, patients were required to visit the hospital every 3 months during the initial 2 years and then once every 6 months.

### Statistical analysis

In the current study, we used individual patients who received neoadjuvant CRT with subsequent surgery as the unit of analysis. First, we used univariate logistic regression to select significant risk factors related to pCR. In the forecasting models, the dependent variable was the probability of pCR, and the independent variables were the significant risk factors.

Second, the data set was randomly segmented into training and testing sets, comprising 70% and 30% of the whole data set, respectively. From a probabilistic perspective related to forecasting models, this randomisation was a form of statistical sampling (e.g. Monte Carlo sampling). We used the training set to construct the forecasting models. The independent variables fitted to the forecasting models were the significant risk factors, and the dependent variable was the outcome (pCR probability). Upon completing training, the forecasting model was exposed to the testing set, and the model outputs were calculated for each testing set. Additionally, for cross‐validation, data from 34 new patients were used to construct the validation set for the prediction of pCR in patients with LARC after neoadjuvant CRT.

Third, the performance indices including sensitivity, 1-specificity, positive predictive value (PPV), negative predictive value (NPV), accuracy, and area under the receiver operating characteristic curve (AUROC) were employed to evaluate the accuracy of the models. Bootstrapping with 1,000 replications was also performed to further amplify the training, testing, and validation data sets to reduce variability in assessments of model performance.

Finally, a global sensitivity analysis was conducted to evaluate the relative significance of input variables in the prediction models; these variables were ranked by their importance. The network error ratio, the sum of squared residuals, represented the global sensitivity of the input variables against the output variables. In general, a variable sensitivity ratio (VSR) of ≤ 1 demonstrates that the variable decreased predictive performance and should be removed. STATISTICA 13.0 (StatSoft, Inc., Tulsa, OK, USA) was used for statistical analyses.

### Ethics approval statement

The study protocol was established according to the ethical principles of the Declaration of Helsinki and was approved by the Institutional Review Board of Kaohsiung Medical University Hospital (KMUHIRB-E (II)-20190280). Each patient provided written informed consent.

## Results

### Patient characteristics

Two hundred seventy patients with LARC were enrolled for analysis. The training and validation cohorts had 236 and 34 patients, respectively (Table 1). The median post-CRT CEA level was 2.2 ng/mL (range 0.48 to 197.5). Accordingly, the cut-off value of post-CRT CEA level was 2 ng/mL. In the training and validation cohorts, respectively 23.7% and 20.6% of patients achieved pCR following CRT (*P* = 0.162).

**Table 1:** Patient characteristics.

Variables	The training cohort mean ± SD/N (%)	The validation cohort mean ± SD/N (%)
**Number of patients**	236	34
**Patient attributes**		
Gender		
Female	82 (34.7)	12 (35.3)
Male	154 (65.3)	22 (64.7)
Age	62.1 ± 11.5	62.8 ± 12.2
**Clinical attributes**		
Chemotherapy		
Fluoropyrimidine	95 (40.3)	15 (44.1)
FOLFOX	141 (59.7)	19 (55.9)
Tumor location		
Low/middle	141 (59.7)	20 (58.8)
Upper	95 (40.3)	14 (41.2)
Clinical T stage		
T2	13 (5.5)	2 (5.9)
T3	184 (78)	27 (79.4)
T4	39 (16.5)	5 (14.7)
Clinical N stage		
N0	36 (15.3)	6 (17.7)
N1	145 (61.4)	20 (58.8)
N2	55 (23.3)	8 (23.5)
TNM stage		
II	36 (15.3)	6 (17.6)
III	200 (84.7)	28 (82.4)
Tumor grade		
Well differentiation	16 (6.8)	2 (5.8)
Moderate differentiation	212 (89.8)	31 (91.3)
Poor differentiation	8 (3.4)	1 (2.9)
Pre-CRT CEA (ng/mL)		
≦ 5	144 (61)	20 (58.8)
> 5	92 (39.0)	14 (41.2)
Anemia		
Hb (g/dL)≦ 10	76 (32.2)	10 (29.4)
Hb (g/dL) > 10	160 (67.8)	24 (70.6)
Diarrhea		
Grade 0–1	102 (43.2)	14 (41.2)
Grade 2–3	134 (56.8)	20 (58.8)
Urinary symptoms		
Grade 0–1	218 (92.4)	31 (91.2)
Grade 2–3	18 (7.6)	3 (8.8)
Dermatitis		
Grade 0–1	166 (70.3)	24 (70.6)
Grade 2–3	70 (29.7)	10 (29.4)
Leukopenia		
WBC≦ 3,000 (/uL)	65 (27.5)	11 (32.4)
WBC > 3,000 (/uL)	171 (72.5)	23 (67.6)
RT dose (cGy)		
5,040	11 (4.7)	1 (2.9)
5,000	181 (76.7)	27 (79.5)
4,500	44 (18.6)	6 (17.6)
RT-surgery interval		
≦8 weeks	81 (34.3)	14 (41.2)
> 8 weeks	155 (65.7)	20 (58.8)
Post-CRT CEA (ng/mL)		
≦ 2	90 (38.1)	13 (38.2)
> 2	146 (61.9)	21 (61.8)
Treatment response		
pCR	56 (23.7)	7 (20.6)
Non-pCR	180 (76.3)	27 (79.4)

*CEA* carcinoembryonic antigen, *CRT* chemoradiotherapy, *FOLFOX* fluorouracil, leucovorin, and oxaliplatin, *Hb* hemoglobin, *pCR* pathological complete response, *SD* standard deviation, *RT* radiation therapy, *WBC* white blood cell.

### Study characteristics

Table 2 presents the training data set’s pCR odds ratio (OR). The univariate analysis indicated that pCR occurrence in patients with LARC after neoadjuvant CRT was significantly associated with gender, age, tumor location, type of chemotherapy, clinical tumor stage, clinical nodal stage, tumor-node-metastasis stage, tumor grade, post-CRT CEA level, anemia, diarrhea, urinary symptoms, dermatitis, leukopenia, radiation therapy dose, and the radiation to surgery interval (*P* < 0.01). As a result, the significant variables were further analyzed in the forecasting models.

**Table 2:** The univariate analysis of logistic regression model using selected risk factors related to pathological complete response (N = 236).

Variables	OR	95% C.I	P-value
**Gender**			
Male vs. female	3.53	2.41–5.17	< 0.001
**Age**	1.02	1.01–1.02	< 0.001
**Chemotherapy**			
FOLFOX vs. fluoropyrimidine	2.53	1.75–3.64	< 0.001
**Tumor location**			
Upper vs. low/middle	4.28	2.56–7.15	< 0.001
**Clinical T stage**			
T2 vs. T3	2.92	2.09–4.06	< 0.001
T2 vs. T4	6.80	2.66–17.4	< 0.001
**Clinical N stage**			
N0 vs. N1	3.68	2.47–5.47	< 0.001
N0 vs. N2	4.00	2.07–7.75	< 0.001
**TNM stage**			
II vs. III	3.76	2.68–5.29	< 0.001
**Tumor grade**			
Well differentiation vs. moderate differentiation	3.00	2.20–4.09	< 0.001
Well differentiation vs. poor differentiation	3.68	2.40–6.97	< 0.001
**Pre-CRT CEA (ng/mL)**			
≦ 5 vs. > 5	4.75	2.77–8.14	< 0.001
**Anemia**			
Grade 0–1 vs. grade 2–3	3.32	2.30–4.80	< 0.001
**Diarrhea**			
Grade 0–1 vs. grade 2–3	2.62	1.80–3.83	< 0.001
**Urinary symptoms**			
Grade 0–1 vs. grade 2–3	8.00	1.84–34.79	0.006
**Dermatitis**			
Grade 0–1 vs. grade 2–3	3.67	2.07–6.49	< 0.001
**Leukopenia**			
Grade 0–1 vs. grade 2–3	2.89	2.05–4.07	< 0.001
**RT-dose (cGy)**			
5,000 vs. 4,500	2.69	1.94–3.74	< 0.001
5,040 vs. 4,500	7.80	3.07–19.79	< 0.001
**RT-surgery interval**			
> 8wk vs. ≦8wk	2.44	1.73–3.46	< 0.001
**Post-CRT CEA (ng/mL)**			
≦ 2 vs. > 2	1.58	0.86–2.88	< 0.001

*CEA* carcinoembryonic antigen, *CI* confidence interval, *CRT* chemoradiotherapy, *FOLFOX* fluorouracil, leucovorin, and oxaliplatin, *Hb* hemoglobin, *OR* odds ratio, *pCR* pathological complete response, *RT* radiation therapy, *WBC* white blood cell.

### Comparisons between these forecasting models

The differences in patient attributes, clinical attributes, and pCR occurrence between the training and testing data sets were insignificant (data not shown). Consequently, samples from these two data sets could be compared to improve the reliability of the validation data sets. ANN-based approaches provide three-layer networks and the relative weights of neurons used for pCR prediction. The ANN 16-4-1 model contains 16 input neurons, 4 hidden neurons, 1 bias neuron in the hidden layer, and 1 output neuron. Table 3 presents comparisons between the training and testing data sets indicating that the ANN model outperformed the KNN, SVM, NBC, and MLR models with respect to sensitivity, 1-specificity, PPV, NPV, accuracy, and AUROC. For cross‐validation, data from 34 newly enrolled patients were used to construct the validation data set for pCR prediction; the ANN model remained the most accurate (Table 4).

**Table 3:** Comparison of 1,000 pairs of prediction models for predicting pathological complete response.

	Sensitivity	1-Specificity	PPV	NPV	Accuracy	AUROC
**Training dataset (n = 165)**						
ANN	0.93	0.84	0.87	0.90	0.87	0.79
KNN	0.81	0.64	0.86	0.64	0.78	0.72
SVM	0.91	0.57	0.85	0.57	0.64	0.73
NBC	0.91	0.49	0.75	0.87	0.75	0.50
MLR	0.90	0.47	0.83	0.39	0.80	0.79
**Testing dataset (n = 71)**						
ANN	0.94	0.87	0.89	0.88	0.86	0.81
KNN	0.89	0.49	0.87	0.46	0.84	0.72
SVM	0.90	0.82	0.85	0.71	0.85	0.74
NBC	0.90	0.85	0.82	0.75	0.78	0.51
MLR	0.84	0.61	0.88	0.69	0.85	0.77

*ANN* artificial neural network, *KNN* K nearest neighbor, *SVM* support vector machines, *NBC* Naive Bayes classifier, *MLR* multiple logistic regression, *PPV* positive predictive value, *NPV* negative predictive value, *AUROC* area under the receiver operating characteristic.

**Table 4:** Comparative performance indices of prediction models when using 34 new validation datasets to predict pathological complete response.

	Sensitivity	1-Specificity	PPV	NPV	Accuracy	AUROC
ANN	0.94	0.80	0.89	0.87	0.88	0.84
KNN	0.80	0.67	0.87	0.60	0.80	0.74
SVM	0.91	0.76	0.86	0.72	0.71	0.76
NBC	0.90	0.53	0.79	0.84	0.80	0.63
MLR	0.88	0.79	0.84	0.49	0.83	0.77

*ANN* artificial neural network, *KNN* K nearest neighbor, *SVM* support vector machines, *NBC* Naive Bayes classifier, *MLR* multiple logistic regression, *PPV* positive predictive value, *NPV* negative predictive value, *AUROC* area under the receiver operating characteristic.

### Significant predictors in the ANN model

We used the training data sets to compute the VSR for the ANN model. The global sensitivity analysis demonstrated that the most sensitive variable for predicting pCR occurrence in patients with LARC after neoadjuvant CRT was post-CRT CEA levels (VSR = 1.57), followed by intervals between radiation and surgery (VSR = 1.50), types of chemotherapy (VSR = 1.45), clinical nodal stages (VSR = 1.37), and clinical tumor stages (VSR = 1.32) (Table 5). All VSR values in the current study exceeded 1, indicating that the network operated better when we considered all variables.

**Table 5:** Global sensitivity analysis of the ANN model in predicting pathological complete response.

	Rank 1st	Rank 2nd	Rank 3rd	Rank 4th	Rank 5th
Variables	Post-CRT CEA	RT-surgery interval	Chemotherapy regimen	Clinical N stage	Clinical T stage
VSR	1.57	1.50	1.45	1.37	1.32

*ANN* artificial neural network, *CEA* carcinoembryonic antigen, *CRT* chemoradiotherapy, *RT* radiation therapy, *VSR* variable sensitivity ratio.

## Discussion

We used performance indices to compare the forecasting models with respect to their accuracy in predicting pCR occurrence in patients with LARC after neoadjuvant CRT. Overall, the ANN model exhibited higher accuracy than did the KNN, SVM, NBC, and MLR models. When we used actual validation data sets to compare performance among forecasting models based on pCR occurrence, the ANN model significantly outperformed the KNN, SVM, NBC, and MLR models, which were constructed using the same limited number of clinical parameters.

Recent studies have consistently demonstrated the ANN model’s superiority relative to the KNN, SVM, NBC, or MLR models^22–24^. Furthermore, statistical analyses have proven the advantages of the ANN model^23^. In particular, the high fault tolerance of ANN models facilitates accurate and appropriate processing of incomplete or noise-added inputs. In addition, nonnormally distributed and highly correlated data can be used to develop nonlinear and linear ANN models, with extensive application in medical big data analysis. Clinical studies have commonly used ANN models for prognosis prediction^11,22,24^. This study’s comparison of various models indicated that the ANN model had the best performance in terms of expanding the set of predictive variables; this facilitates evaluation of the effectiveness of research methods and enables comprehensive prediction of pCR occurrence. For other cancer types, the established model can be used to predict clinical outcomes or events.

Machine learning has been widely applied for predicting responses to cancer therapy. Bibault et al*.* used deep learning combined with clinical and radiomic features to predict pCR in patients with LARC following neoadjuvant CRT. They demonstrated that the deep neural network achieved higher accuracy than the linear regression and SVM models did^25^. Metser et al. evaluated the correlation between radiomic features and pCR by using machine learning algorithms and revealed that the classifier trained on pretreatment positron emission tomography scans had an accuracy of 92.8% in predicting pCR to CRT in patients with LARC^26^. Furthermore, machine learning for treatment response prediction has been used for patients with cancer of the head and neck, breast, lung, and prostate^15,22,27,28^. Many studies have demonstrated the favorable performance of machine learning for treatment response prediction related to different cancer types. Our results supported the high accuracy of the ANN model in predicting the efficacy of CRT for LARC.

In the current study, the ANN model exhibited higher accuracy than did the MLR model, a traditional and widely used statistical model in medicine. Growing evidence indicates that overall, machine learning models have higher accuracy in predicting oncologic outcomes than do logistic regression models. According to Faradmal et al., ANN model accuracy was higher than that of the logistic regression model for predicting breast cancer recurrence^29^. Similarly, Alabi et al. compared an ANN model with a logistic regression model based on their prediction of locoregional recurrence in patients with early oral tongue carcinoma, and the ANN model was superior^28^. In the aforementioned studies, machine learning methods exhibited superior accuracy than traditional methods.

A global sensitivity analysis was performed to evaluate the value of significant predictors affecting pCR occurrence. We determined post-CRT CEA level to be the most important predictor of pCR occurrence in patients with LARC after neoadjuvant CRT. CEA level has been commonly evaluated in colorectal cancer–related predictions. Several studies have highlighted the predictive value of post-CRT CEA levels in patients with LARC treated with neoadjuvant CRT. Peng et al*.* revealed that a post-CRT CEA level of ≤ 2 ng/mL was an independent predictors of pCR (OR 1.579; 95% confidence interval [CI] 1.02 to 2.43; *P* = 0.038)^30^. Yang et al*.* identified a post-CRT CEA level of ≤ 2.61 ng/mL as being significantly associated with pCR (OR 0.605; 95% CI 0.41 to 0.89; *P* = 0.011) and improved overall survival^31^. Kleiman et al*.* reported a significant correlation between decreased post-CRT CEA levels and pCR occurrence (OR 1.74; 95% CI 1.06 to 3.81)^32^. A possible explanation might be that decreased post-CRT CEA levels indicate prominent effects of CRT and consequently favorable tumor regression. However, the literature on the exact mechanism remains scarce.

Because radiation-induced necrosis requires time to develop, a prolonged interval between radiation and surgery potentially increases pCR occurrence. In the current study, a radiation-surgery interval > 8 weeks was associated with high pCR rates. The association between longer intervals and pCR occurrence has been studied in several retrospective cohorts, with inconsistent findings. Kalady et al*.* and Probst et al*.* have demonstrated that intervals > 8 weeks are associated with increased pCR occurrence^33,34^, but Stein et al*.* and Sun et al*.* have reported the opposite result^35,36^. In our previous study, we demonstrated that a longer CRT-surgery interval was associated with increased pCR rates^7^. Several randomized trials have been published to resolve this inconsistency. Two randomized trials by Akgun et al*.* and Terzi et al*.* have demonstrated that pCR rates are higher for long intervals (> 8 weeks) than for short intervals, although both intervals have exhibited similar rates in postoperative mortality and morbidity^37,38^. However, the GRECCAR-6 trial revealed no significant difference between long (11 weeks) and short intervals (7 weeks) concerning pCR occurrence, although greater complications and difficulties in surgery were observed for participants with an 11-week interval^39^. More data are required to determine which interval best increases pCR occurrence.

To enhance response to CRT, several chemotherapeutic drugs were added to standard fluoropyrimidine-based CRT. Two randomized trials have reported an increase in pCR after the addition of oxaliplatin to CRT^5,6^, but other trials have revealed no such increase^4,40,41^. To resolve this inconsistency, Yang et al*.* reviewed the published randomized trials and demonstrated that the addition of oxaliplatin to CRT significantly increased pCR rates (risk ratio 1.24; 95% CI 1.02 to 1.51; *P* = 0.03)^42^. Our previous study revealed that FOLFOX-based CRT resulted in improved pCR rates relative to fluoropyrimidine-based CRT^7^. In the current study, we also determined that FOLFOX-based CRT constituted an independent predictor of pCR in machine-learning prediction models.

In agreement with our results, several studies have demonstrated that having clinically node-negative rectal cancer is independently associated with an increase in pCR occurrence^43–45^. Our previous study reviewed 236 patients with LARC undergoing neoadjuvant CRT with subsequent surgery. According to the results, pCR rates in clinically node-negative diseases were three times higher than in node-positive diseases (OR 3.2; 95% CI 1.27 to 8.41; *P* = 0.013)^46^. Based on these studies, clinical node positivity may indicate more advanced disease, which results in poor response to CRT. Therefore, watch-and-wait treatment is likely to be suitable for patients with clinically node-negative rectal cancer.

In this study, the ANN model identified clinical T4 as an independent predictor for the absence of pCR. This finding is consistent with those of other studies demonstrating that an advanced tumor stage is associated with unfavorable tumor regression^43–45^. Despite contradictory findings on the association between clinical tumor stage and pCR occurrence^33,47^, clinical experience suggests that a highly advanced tumor stage is associated with highly aggressive tumor behavior, indicating lower sensitivity to CRT.

In addition to improving the analysis of variance in the correlation between clinical parameters and pCR occurrence, predictive models have broad clinical applications. The methods used in this study can be applied to investigate the effectiveness of other treatment methods, and the quality of care can thus be improved. Because the proposed ANN model exhibited high accuracy in predicting pCRs, the model can help clinicians identify which patients can benefit from watch-and-wait treatment after neoadjuvant CRT. More studies are required to confirm the reliability of the ANN model and to clarify whether it can be used to effectively predict clinical outcomes and optimize cancer treatment.

This study had some limitations. First, MRI features were not assessed: comparisons are of limited validity because of incompleteness in MRI data. Second, the focus on pCR as the endpoint of this prediction model potentially limits the overall clinical utility of the ANN model to a small subset of patients who have a high likelihood of achieving pCR. Third, we only ran forecasting models to predict pCR in patients with LARC after neoadjuvant CRT. Because of the robust magnitude and statistical significance of the effects in the current study, we contend these limitations did not compromise the validity of the results.

## Conclusions

Relative to the KNN, SVM, NBC, and MLR models, this study’s ANN model was more accurate in predicting pCR in patients with LARC after neoadjuvant CRT, at higher overall performance indices. Those giving preoperative consultations can use this study’s predictors to educate candidates on choices of LARC surgery in terms of health outcomes and the expected prognosis. These findings can serve as a vital and empirical foundation for improving the quality of life of patients with LARC due to the omission of radical surgery.

## Data Availability

All data generated or analyzed during this study are included in this published article.
